# Molecular regulation of PPARγ/RXRα signaling by the novel cofactor ZFP407

**DOI:** 10.1371/journal.pone.0294003

**Published:** 2024-05-23

**Authors:** Alyssa Charrier, Jeremiah Ockunzzi, Leighanne Main, Siddharth V. Ghanta, David A. Buchner

**Affiliations:** 1 Department of Genetics and Genome Sciences, Case Western Reserve University, Cleveland, Ohio, United States of America; 2 Department of Population and Quantitative Health Sciences, Case Western Reserve University, Cleveland, Ohio, United States of America; 3 Department of Biochemistry, Case Western Reserve University, Cleveland, Ohio, United States of America; Tokyo University of Agriculture, JAPAN

## Abstract

Cofactors interacting with PPARγ can regulate adipogenesis and adipocyte metabolism by modulating the transcriptional activity and selectivity of PPARγ signaling. ZFP407 was previously demonstrated to regulate PPARγ target genes such as *GLUT4*, and its overexpression improved glucose homeostasis in mice. Here, using a series of molecular assays, including protein-interaction studies, mutagenesis, and ChIP-seq, ZFP407 was found to interact with the PPARγ/RXRα protein complex in the nucleus of adipocytes. Consistent with this observation, ZFP407 ChIP-seq peaks significantly overlapped with PPARγ ChIP-seq peaks, with more than half of ZFP407 peaks overlapping with PPARγ peaks. Transcription factor binding motifs enriched in these overlapping sites included CTCF, RARα/RXRγ, TP73, and ELK1, which regulate cellular development and function within adipocytes. Site-directed mutagenesis of frequent PPARγ phosphorylation or SUMOylation sites did not prevent its regulation by ZFP407, while mutagenesis of ZFP407 domains potentially necessary for RXR and PPARγ binding abrogated any impact of ZFP407 on PPARγ activity. These data suggest that ZFP407 controls the activity of PPARγ, but does so independently of post-translational modifications, likely by direct binding, establishing ZFP407 as a newly identified PPARγ cofactor. In addition, ZFP407 ChIP-seq analyses identified regions that did not overlap with PPARγ peaks. These non-overlapping peaks were significantly enriched for the transcription factor binding motifs of TBX19, PAX8, HSF4, and ZKSCAN3, which may contribute to the PPARγ-independent functions of ZFP407 in adipocytes and other cell types.

## Introduction

More than one-third of adults in the United States are obese [1]. These individuals demonstrate an increased risk for premature mortality given their higher incidence of comorbidities such as type 2 diabetes (T2D) and cardiovascular disease [2]. Although treatments for these conditions exist, safely maintaining a healthy body weight and long-term glucose homeostasis remains a challenge for obese individuals [3]. As key regulatory cells in metabolic signaling and the primary site for lipid storage, adipocytes are central to the pathogenesis of obesity and T2D, displaying promise as targets for therapeutics modulating metabolic processes [4]. Within adipocytes, the peroxisome proliferator-activated receptor (PPAR) family of nuclear receptors, and especially PPARγ, regulate genes involved in glucose homeostasis, adipogenesis, and lipid metabolism [5]. Through an obligate heterodimerization complex with Retinoid X receptors (RXRs), PPARγ recruits additional cofactors necessary to coordinate metabolic processes such as insulin responsive glucose uptake and lipolysis [6].

PPARγ is both necessary and sufficient to regulate adipocyte differentiation and metabolism [7]. PPARγ ligands such as rosiglitazone and pioglitazone, both thiazolidinediones (TZDs), have been utilized as T2D therapeutics on account of their ability to improve insulin responsiveness and lower hyperglycemia, although clinical complications such as increased risk of bone fractures and bladder cancer have limited their use [8]. TZD-induced activation of PPARγ upregulates transcription of genes in key metabolic pathways, such as the *GLUT4* glucose transporter, lipoprotein lipase (*LPL*), and adipocyte fatty acid binding protein (*FABP4*), among others [9]. In addition to their actions in adipose tissue, TZDs improve insulin resistance in skeletal muscle and liver tissue. However, as PPARγ is more highly expressed in adipocytes, this may be related to endocrine signaling involving free fatty acids and tumor necrosis factor-alpha (TNF-α) originating in adipose tissue [10, 11].

Within adipocytes, PPARγ activity displays three primary mechanisms of action. 1) Ligand-independent repression is observed when PPARγ activation inhibits c-Jun N-terminal kinase (JNK) MAPK activity, thereby downregulating gene expression of MAPK targets [12]. 2) PPARγ exhibits both agonist-dependent activation or repression, as in the case of peroxisome proliferator-activated receptor g coactivator 1α (PGC-1α) or nuclear receptor corepressor 1 (NCoR1) biding, respectively [13]. 3) PPARγ activity is also modulated by post-translational modifications such as phosphorylation, acetylation, glycosylation, SUMOylation, and ubiquitination [13]. However, despite decades of study, much of the specific mechanisms underlying PPARγ regulation remains unknown [7, 14]. For example, it remains unclear why only a limited number of PPARγ DNA binding sites in adipocytes appear to affect transcription [7, 15–17]. This is likely the result of the combinatorial effects of PPARγ with other uncharacterized transcriptional regulators, which may represent opportunities for novel therapeutics in T2D and obesity treatment by activating subsets of PPARγ targets that improve insulin sensitization without the negative side-effects associated with TZD treatment.

ZFP407 is one such poorly understood protein that has been previously demonstrated to positively regulate PPARγ [18]. Utilizing cultured adipocytes, ZFP407 was shown to have broad transcriptional effects on PPARγ signaling, including its regulation of *GLUT4* mRNA, which is directly tied to insulin responsiveness in adipose and muscle tissue [19]. Further *in vivo* studies in mice demonstrated ZFP407 overexpression improves glucose homeostasis [19], while its deficiency causes lipodystrophy and exacerbates insulin resistance [20]. Collectively, these studies suggest that ZFP407 is a key regulatory molecule of PPARγ, with unique non-redundant functionality in PPARγ signaling.

Recent studies have demonstrated the impact of context dependence and combinatorial transcription factor action on transcriptional regulation of gene expression [21–26]. With regards to T2D and obesity treatments, this underscores the importance of identifying and characterizing new regulatory cofactors of PPARγ, particularly ones such as ZFP407, which impacts both transcriptional regulatory networks as well as adipocyte development and homeostasis. Utilizing cellular models in this study, we sought to improve the mechanistic understanding of ZFP407’s molecular regulation of PPARγ signaling to better elucidate its role in adipocyte physiology.

## Materials and methods

### Materials

Insulin, dexamethasone, and 3-isobutyl-1-methylxanthine, and fetal bovine serum (FBS) were obtained from Sigma Aldrich (USA). Dulbecco’s Modified Eagle’s Medium (DMEM), L-Glutamine/Pen/Strep, 0.05% Trypsin-EDTA, and 0.25% Trypsin-EDTA were obtained from Life Technologies (USA).

### Cell culture

293T cells were cultured in DMEM with 10% FBS and 1x L-Glutamine/Pen/Strep and passaged with 0.05% Trypsin-EDTA. 3T3-L1 cells were cultured, passaged, and differentiated as previously described [27].

### Immunostaining and subcellular fractionization

293T cells were seeded at a density of 5x10^5^ cells onto Poly-D-lysine coated coverslips in 12-well cell culture dishes and incubated overnight at 37°C in 5% CO_2_. Cultured cells were then transfected with either empty vector control, *ZFP407* + empty vector control, empty vector control + *PPARγ* or *PPARγ* + *ZFP407* plasmids using Lipofectamine 3000 (ThermoFisher Scientific, Waltham, MA, USA) according to manufacturer’s protocol. Transfected plasmids encoded *PPARγ* (Addgene #8862), *ZFP407* (cat. #: MR214555, Origene Technologies, USA), or an empty vector control (*pRK5-Myc*, Clontech, USA). Cells were briefly fixed with 1:1 dilution of methanol and acetone at -20°C and rinsed with TBS. Double immunofluorescent staining of cell-culture slides for ZFP407 and PPARγ was performed using anti-mouse c-Myc (9E10) (5ug/ml), (Santa Cruz Biotechnology, USA) and anti-rabbit PPAR (D69) (1:100, Cell Signaling Technology, USA) followed by incubation with Alexa-fluor 488 goat-anti-mouse (1:1000) and 568 goat-anti rabbit (1:1000) for 1 hour at room temperature. Cell-covered coverslips were mounted onto slides with ProLong Diamond Antifade Mountant with DAPI (ThermoFisher Scientific, USA).

Subcellular fractionation was performed using the Nuclear Complex Co-IP kit (Active Motif, USA) according to manufacturer’s protocol until isolation of the nucleus. At this step, the pellet (nuclear fraction) and the supernatant (cytoplasmic fraction) were independently analyzed by western blot.

### Co-IP and western blotting

Co-immunoprecipitation (Co-IP) was performed using the Nuclear Complex Co-IP kit (Active Motif, USA) and Dynabeads Protein G for Immunoprecipitation according to manufacturer’s protocol (Invitrogen, USA), and western blotting was performed as previously described [18]. Anti -PPARγ (cat.#: 2430 and 2443), anti-RXRα (cat.#: 3085), and anti-IgG (cat.#: 2729) antibodies were obtained from Cell Signaling (USA). Anti-GAPDH (cat.#: MA5-15738) antibody was obtained from Thermo Fischer Scientific (USA). A custom anti-ZFP407 antibody was generated in rabbits against the C-terminal 149 amino acids of the mouse ZFP407 protein (Proteintech Group, USA), as previously described [19]. Goat anti-rabbit (cat.#: 31460) and goat anti-mouse (cat.#: 31430) secondary antibodies were obtained from Thermo Fisher Scientific (USA). Primary antibodies were diluted 1:1,000, while secondary antibodies were diluted 1:10,000.

### Chromatin immunoprecipitation (ChIP)-seq assay and motif analysis

ChIP was carried out in 3T3-L1 differentiated adipocytes using 30 ug of cell chromatin. ChIP DNA was processed using the Illumina ChIP-Seq kit according to manufacturer’s protocols (Illumina, USA) and sequenced to a depth of 11.5 million reads. The FASTX-Toolkit v0.0.13 was used to quality filter reads using a quality score cutoff of 20. These reads were aligned to the mouse genome (mm9) using Bowtie2 v2.0.6 and any reads with at least one mismatch were discarded. PCR duplicates were also removed using SAMtools v1.3, leaving 9.2 million reads. The MACS2 algorithm [28] identified 7,313 peaks (q-value < 0.001) using the narrow filter and excluding ENCODE blacklist sites. Control bias-corrected bedGraphs, generated by MACS2 were converted to bigWIGs and used for genome browser visualization. BED files containing genomic coordinates for ChIP-seq peaks were extracted from the UCSC genome browser [29] post visualization and the GRanges R package [30] was used to segregate the top 1,000 ZFP407 ChIP-seq peaks (by q-score) based on genomic co-occupancy with PPARγ, CEBPα, and RXRα ChIP-seq peaks obtained from previous datasets [17, 31, 32] into overlapping and non-overlapping groups. Using the top 1,000 ZFP407 peaks, ZFP407 peaks overlapping with PPARγ ChIP-seq peaks, those not overlapping with PPARγ ChIP-seq peaks, and ZFP407 peaks not overlapping with any other of the above mentioned dataset’s peaks, motif enrichment analysis was performed using Hypergeometric Optimization of Motif EnRichment (HOMER) [33] to determine *de novo* binding motifs enriched in each peak population. Motif searches were performed using the default region size of 200 against the mm9 genome using the genomic background option. The HOMER function *findMotifsGenome*.*pl* was employed on each set of segregated peaks for motifs of lengths ranging from 8 to 25 bps. HOMER motif analysis p-values were calculated by the program itself, utilizing the suggested cut-off of p<1x10^-12^ to identify significantly enriched motifs with the lowest likelihood of false positives. Nucleotide probability matrices from HOMER *de novo* motifs with p value ≤ 1x10^-12^ were individually run through the 10^th^ release (2024) of the JASPAR Database CORE collection [34] for best matches to known TF binding motifs [31–33]. ZFP407 ChIP-Seq data is available through NCBI’s GEO and can be retrieved with the accession number GSE245861.

Recently, a binding motif was proposed for ZNF407, the human ortholog of ZFP407 [35]. Because this TF was predicted to have high evolutionary conservation [36], we performed a further assessment of genomic regions corresponding to our ZFP407 ChIP-seq peaks to probe for motifs consistent with the human ZNF407 binding site motif. Genomic coordinates for the top 1,000 ZFP407 ChIP-seq peaks were visualized via the UCSC Genome Browser as a BED file and genomic sequences for each region were extracted. A position frequency probability matrix for the novel ZNF407 binding site motif proposed by Pratt et al. [35] (ZN407.H12CORE.0.P.B) was extracted from the Hocomoco database v12 [37]. Genomic sequences corresponding to ZFP407 ChIP-seq peaks were scanned using the FIMO tool from the MEME suite v5.5.5 [38] to search for instances of the ZNF407 motif, the human ortholog of ZFP407. To test for specificity of potential ZNF TF binding in these sites, five other randomly chosen ZNF motifs with comparable sizes (22–25 bp) were also analyzed using the FIMO tool. The 5 motifs corresponded to ZNF8, ZNF250, ZNF260, ZNF329, and ZNF768.

### PPARγ and ZFP407 site-directed mutagenesis

Using the Q5 Site-Directed Mutagenesis Kit according to manufacturer’s protocol (New England Bio Labs, USA), point mutations were created at multiple sites in PPARγ. Lowercase nucleotides indicate mutated residue sites. Serine 112 was mutated to alanine using a forward primer with the sequence 5’- AGAAC CTGCA gctCC ACCTT ATT -3’ and a reverse primer with the sequence 5’- ACTTT GATCG CACTT TGGTA TTC -3’. Serine 112 was mutated to aspartate using a forward primer with the sequence 5’- AGAAC CTGCA gatCC ACCTT ATTAT TC -3’ and a reverse primer with the sequence 5’- ACTTT GATCG CACTT TGG -3’. Lysine 107 was mutated to arginine using a forward primer with the sequence 5’- AAGTG CGATC aaaCG AGTAG AACCT G -3’ and a reverse primer with the sequence 5’- TGGTA TTCTT GGAGC TTC -3’. Serine 273 was mutated to alanine using a forward primer with the sequence 5’- AACGG ACAAA tGCAC CATTT GTC -3’ and a reverse primer with the sequence 5’- GTCTT TCCTG TCAAG ATCG -3’. Additionally, point mutations were induced at multiple sites in ZFP407. The PPARγ binding motif (amino acids 1980–1984) was mutated from LDALL to ADALA using a forward primer with the sequence 5’- actgg cgTGT GCTGT CACTG AGTTG -3’ and a reverse primer with the sequence 5’- gcatc tgcGG CTGAG GAGTT GTCAG ATG -3’. The coRNR motif (amino acids 2140–2144) was mutated from ISQII to ISQAA using a forward primer with the sequence 5’- GATCT CTCTC Aggcc gctGT AACAG AAGAG CTAGT C -3’ and a reverse primer with the sequence 5’- TCTCC TTCTG ACTCT ACC -3’.

### PPARγ luciferase reporter assay

293T cells were transfected with Lipofectamine 3000 (Life Technologies, USA) according to manufacturer’s protocol (Life Technologies, USA). 3T3-L1 cells were electroporated as previously described [39]. Transfected plasmids encoded *PPARγ* (Addgene #8862), *PPARγ* mutants, *Zfp407* (cat. #: MR214555, Origene Technologies, USA), or an empty vector control (pRK5-Myc, Clontech, USA), as well as the *PPARγ* target gene luciferase reporter plasmid (PPRE) (Addgene #1015). The pRL-SV40 plasmid encoding Renilla luciferase was added for normalization. For 293T cells, 490 ng of plasmid DNA, 100 ug of PPRE reporter, and 10 ng of pRL-SV40 were added per reaction. For 3T3-L1 cells, 99μg of plasmid DNA, 10ng PPRE reporter, and 1μg of pRL-SV40 were added per reaction. Relative luciferase activity was measured 24 h post-transfection with the Dual-Glo Luciferase Assay System (Promega, USA).

### Statistics

Luciferase reporter assays were analyzed via two-tailed Student t-test. ZNF motif frequency was compared using the Crawford-Howell test [40]. A p-value ≤0.05 was considered statistically significant. Data shown represent group means ± SE.

## Results

### Nuclear co-localization of PPARγ and ZFP407

**Fig 1:**
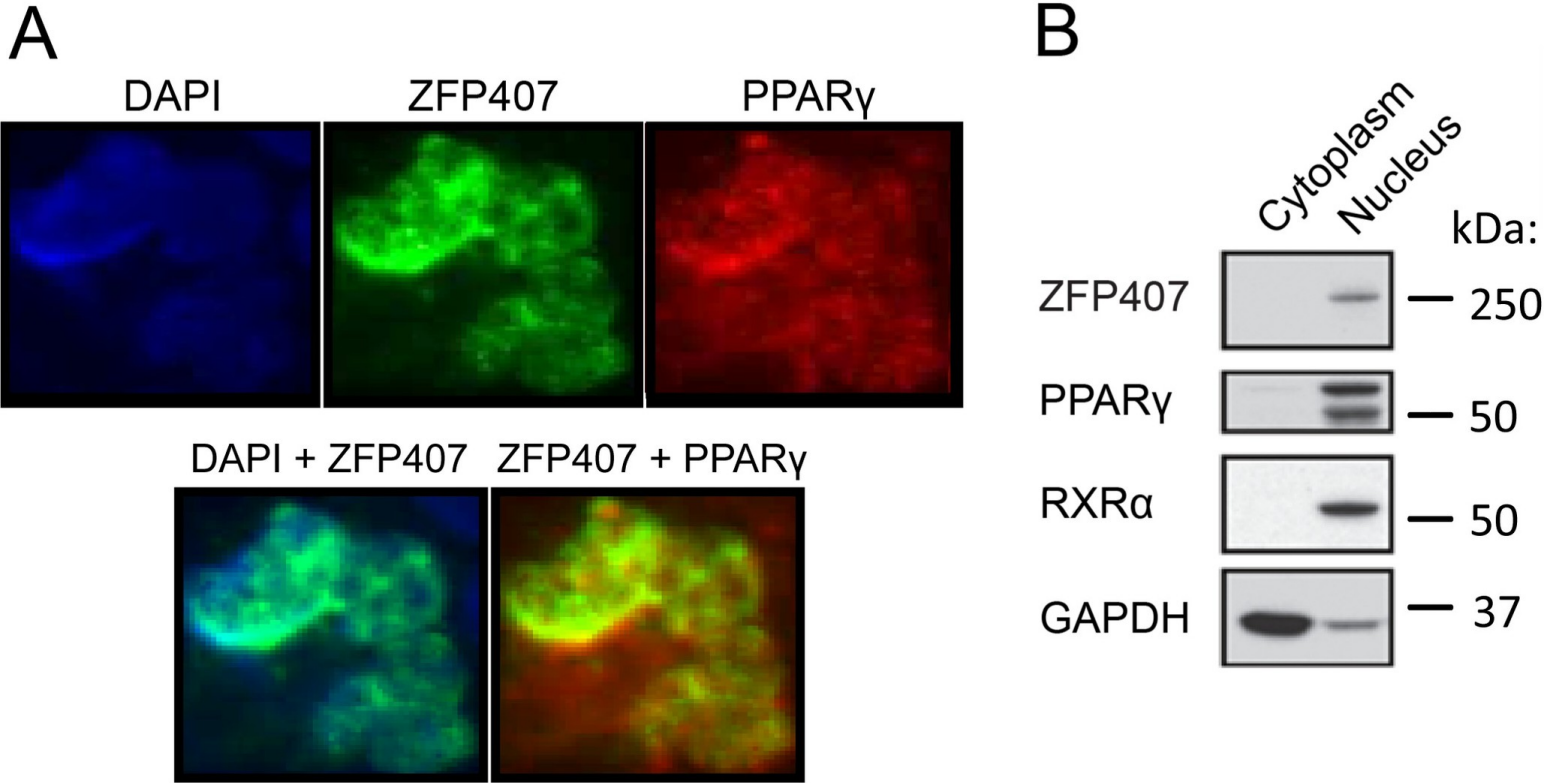
Nuclear localization of ZFP407. (A) Immunostaining of 293T cells co-transfected with plasmids encoding ZFP407 and PPARγ. (B) Subcellular fractionation of 3T3-L1 differentiated adipocytes blotted for ZFP407, PPARγ, RXRα, and GAPDH.

To determine the subcellular localization of ZFP407, and potential co-localization with PPARγ, 293T cells were transfected with plasmids encoding expression cassettes of either ZFP407, PPARγ, or both together. Immunostaining demonstrated localization of both proteins in the nucleus when either transfected individually or co-transfected, with considerable overlap between the two proteins when transfected together (Fig 1A). Co-transfection was repeated in differentiated adipocyte 3T3-L1 cells, after which subcellular fractionation was used to separate the nuclear and cytoplasmic components. Western blotting confirmed ZFP407 and PPARγ localization in the nuclear, but not the cytoplasmic, fraction along with RXRα, another protein localized in the nucleus and a known binding partner of PPARγ [2] (Fig 1B).

### ZFP407 protein interacts with the PPARγ/RXRα protein complex

**Fig 2:**
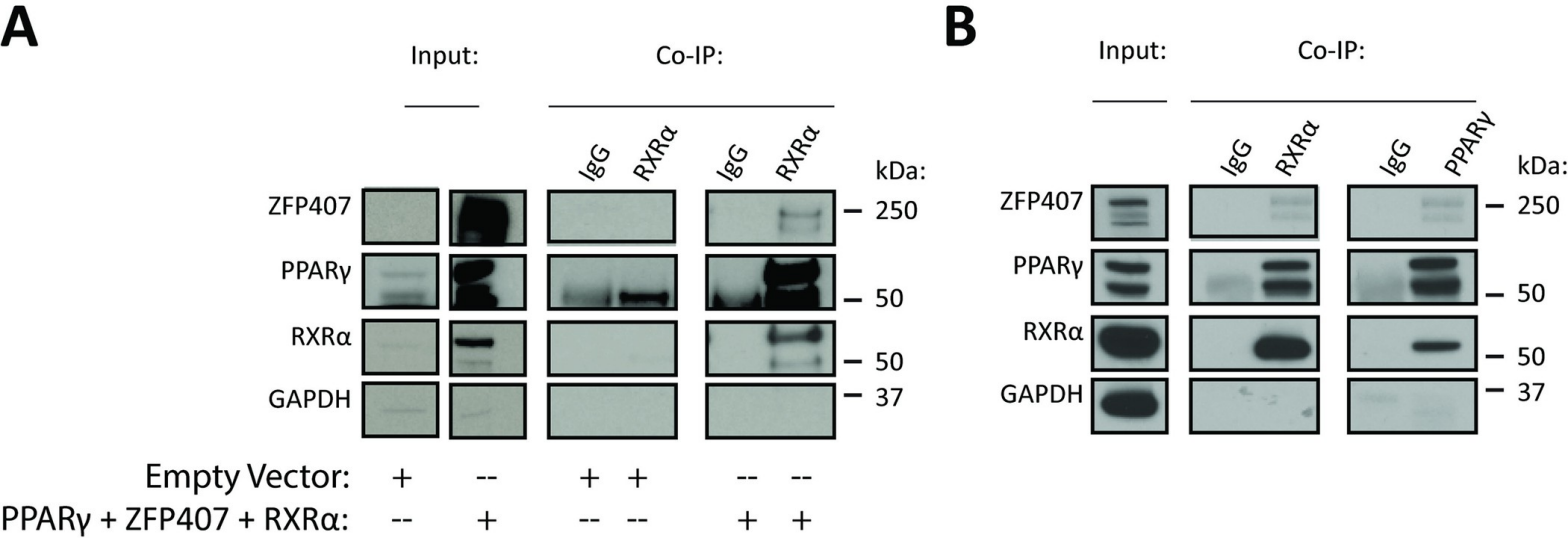
ZFP407 binding in the PPARγ/RXRα protein complex. (A) Co-IP of 293T cells utilizing an anti-RXR antibody performed following transfection of either an empty vector or co-transfection of PPARγ, ZFP407, and RXRα plasmids. (B) Co-IP of 3T3-L1 differentiated adipocyte nuclear extracts using a matched IgG, anti-RXR, or anti-PPARγ antibody.

Given the overlapping subcellular localizations of PPARγ and ZFP407, we next tested whether these proteins were part of the same complex. ZFP407 and PPARγ were both found to interact with the RXRα protein complex in Co-IP experiments performed on nuclear fractions of 293T cells (Fig 2A) using an anti-RXRα pulldown of exogenously expressed proteins. Co-IP was similarly performed for the endogenous proteins using the nuclear fractions of 3T3-L1 differentiated adipocytes. ZFP407 was also found to interact with the endogenous PPARγ/RXRα protein complex, showing specific interactions in pulldowns with both an anti-RXRα and an anti-PPARγ antibody (Fig 2B), indicating ZFP407’s participation in the adipocyte PPARγ/RXRα protein complex.

### Overlap between ZFP407 and PPARγ ChIP-seq peaks

Since ZFP407 is a component of the PPARγ/RXRα protein complex, a significant overlap in chromatin binding sites for these proteins was hypothesized. To test this hypothesis, ChIP-Seq was performed on differentiated 3T3-L1 adipocytes, identifying 7,313 peaks for ZFP407 throughout the genome. On account of the high evolutionary conservation of many ZNF DNA binding motifs across species [36], the proposed human ZNF407 DNA binding motif was searched for within our murine 3T3-L1 ChIP-seq peaks. Within the 785,459 nucleotides spanning the top 1,000 ZFP407 peaks, the FIMO tool uncovered 2,195 motif occurrences of ZNF407 with a p-value < 0.0001. For five other randomly chosen ZNFs with a similar motif length (ZNF8, ZNF250, ZNF260, ZNF329, and ZNF768) the FIMO tool uncovered many fewer motif occurrences, with 45, 75, 63, 382, and 361 potential motif occurrences, respectively, with a p-value < 0.0001 (Table 1). The Crawford-Howell test was used to compare occurrences of the ZNF407 motif to occurrences for the randomly selected zinc-finger motifs of similar length and yielded a p-value of < 0.001, demonstrating significant enrichment specifically of the ZNF407 motif relative to other ZNF motifs. This suggests that the binding motif for murine ZFP407 is evolutionarily conserved and therefore similar to the binding motif for the human ortholog ZNF407, as well as supports the specificity of the ZFP407 ChIP-Seq data.

**Table 1:** Motif occurrences within ZFP407 ChIP-seq peaks.

TF	Hocomoco motif ID	bp Length	Matches within Peak Sequences
ZNF407	ZN407.H12CORE.0.P.C	25	2195
ZNF8	ZNF8.H12CORE.1.P.B	22	45
ZNF250	ZN250.H12CORE.0.P.C	23	75
ZNF260	ZN260.H12CORE.0.P.C	23	63
ZNF329	ZN329.H12CORE.0.P.C	22	382
ZNF768	ZN768.H12CORE.0.P.B	23	361

The FIMO tool was used to probe ZFP407 ChIP-seq peaks for specific occurrences of the indicated motifs.

**Fig 3:**
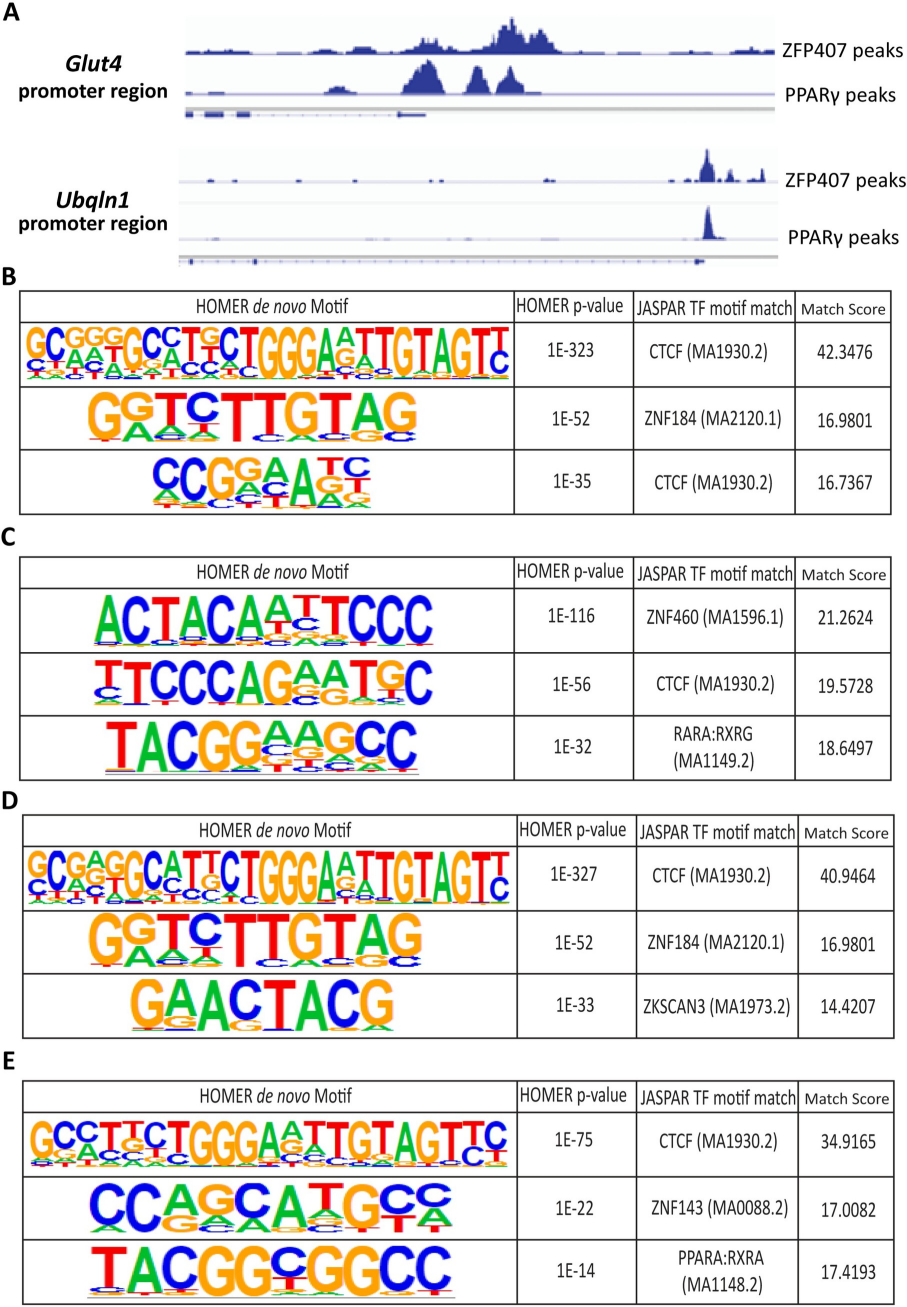
ZFP407 ChIP-seq peaks overlap with PPARγ peaks. (A) Co-occupancy of ZFP407 and PPARγ observed in promoter regions of *Glut4* and *Ubqln1* genes, both of which are PPARγ and ZFP407 target genes. Genomic intron/exon structure for each gene is shown below ChIP data. (B) Top 3 most significant HOMER determined enriched motifs in top 1,000 ZFP407 ChIP-Seq peaks with the JASPAR matched TF binding motifs. (C) Top 3 most significant motifs enriched in peaks overlapping PPARγ ChIP-Seq peaks with their JASPAR matched TF binding motifs. (D) Top 3 motifs enriched in non-overlapping ChIP-Seq sites with their JASPAR matched TF binding motifs. (E) Top 3 most enriched motifs and their JASPAR matched TF binding motifs for ZFP407 peaks not overlapping with PPARγ, CEBPα, or RXRα peaks.

The top 1,000 ZFP407 peaks identified were aligned with a previous PPARγ ChIP-Seq experiment also performed in 3T3-L1 cells [31]. Slightly more than half (50.4%) of all identified ZFP407 peaks overlapped with PPARγ peaks, with even greater overlap (64.8%) within the top 1,000 ZFP407 peaks (Table 2). These included overlapping peaks in the promoter regions of *GLUT4* and *UBQLN1* (Fig 3A), both genes that are regulated by PPARγ and ZFP407 individually [18]. When comparing this PPARγ ChIP-Seq dataset [31] with another published PPARγ ChIP-Seq dataset, also from 3T3-L1 cells [32], only 58.7% of sites from the latter ChIP-Seq experiment were identified in both PPARγ ChIP-Seq datasets. This suggests that the 50.4% overlap, or 64.8% overlap for the top 1,000 peaks, between ZFP407 and PPARγ likely underestimates the degree of overlap when accounting for variance between ChIP-Seq assays examining PPARγ binding in 3T3-L1 cells, as has been noted across multiple studies [32].

**Table 2:** Overlap of ZFP407 ChIP-seq peaks in adipocytes with other TFs.

Overlaps for Top 1000 ZFP407 ChIP-seq Peaks	Number	Percentage
PPARγ	648	64.8%
CEBPα	584	58.4%
RXRα	149	14.9%
Non-overlapping	189	18.9%
**Multiple TF Overlaps with ZFP407**		
PPARγ + CEBPα	339	33.9%
PPARγ + RXRα	22	2.2%
CEBPα +RXRα	9	0.9%
PPARγ + CEBPα + RXRα	100	10.0%
Overlaps for All ZFP407 ChIP-seq Peaks (n = 7,313)	Number	Percentage
PPARγ	3687	50.4%
CEBPα	3126	42.7%
RXRα	1031	14.1%
Non-overlapping	251	3.4%
**Multiple TF Overlaps with ZFP407**		
PPARγ + CEBPα	1359	18.6%
PPARγ + RXRα	262	3.6%
CEBPα +RXRα	113	1.5%
PPARγ + CEBPα + RXRα	486	6.6%

ZFP407 data is from the ChIP-seq experiments described in this manuscript. The PPARγ and CEBPα data are available under the GEO accession number GSE49423. The RXRα data is available under the GEO accession number GSE13511.

In addition to PPARγ, the overlap of ZFP407 and C/EBPα DNA binding peaks were examined. C/EBPα is another key transcriptional regulator in 3T3-L1 cells that is necessary for adipogenesis [41]. In this dataset [31], there was only 36.1% overlap between C/EBPα and PPARγ ChIP-seq peaks and 42.7% overlap between ZFP407 and C/EBPα (Table 2). This suggests ZFP407 may more specifically regulate PPARγ signaling than other transcription factors regulating the expression of genes within the canonical adipogenic pathway.

In order to examine any potential functions of ZFP407 both independent of and relating to PPARγ, we segregated the top 1,000 ZFP407 peaks into those that overlapped with PPARγ ChIP-Seq peaks and those that did not overlap with PPARγ ChIP-Seq peaks (S1 Table), as well as peaks that did not overlap with any ChIP-seq dataset. The motif analysis program HOMER was used to identify enriched TF binding site motifs. When motif analysis was performed on the entire set of top 1,000 ZFP407 peaks, enriched motifs included CTCF, RARα/RXRγ, TBX19, ZNF143, and ZNF46, among others (Fig 3B, S1 Table). Within ZFP407 peaks that overlapped with PPARγ, CTCF and RARα/RXRγ again were identified by JASPAR’s Matrix Align tool, with TP73 and FOXO/ELK1 also demonstrating significant enrichment (Fig 3C, S1 Table). Among the ZFP407 and PPARγ non-overlapping peaks, motifs for CTCF, RARα/RXRγ, TBX19, HSF4, PAX8, and ZKSCAN3 were identified (Fig 3D, S1 Table). For the ChIP-seq peaks that were unique to the ZFP407 dataset, motifs for CTCF, ZNF143, and PPARα/RXRα were identified (Fig 3E). In addition, when performing HOMER analysis on the entirety of ZFP407 ChIP-seq peaks, the PPAR Response Element (PPRE) demonstrated significant enrichment (S1 Table, p = 0.001), consistent with prior results showing ZFP407’s ability to positively regulate PPARγ transcriptional activity towards the PPRE sequence [18].

### PPARγ interaction motifs are required for transcriptional activation by ZFP407

**Fig 4:**
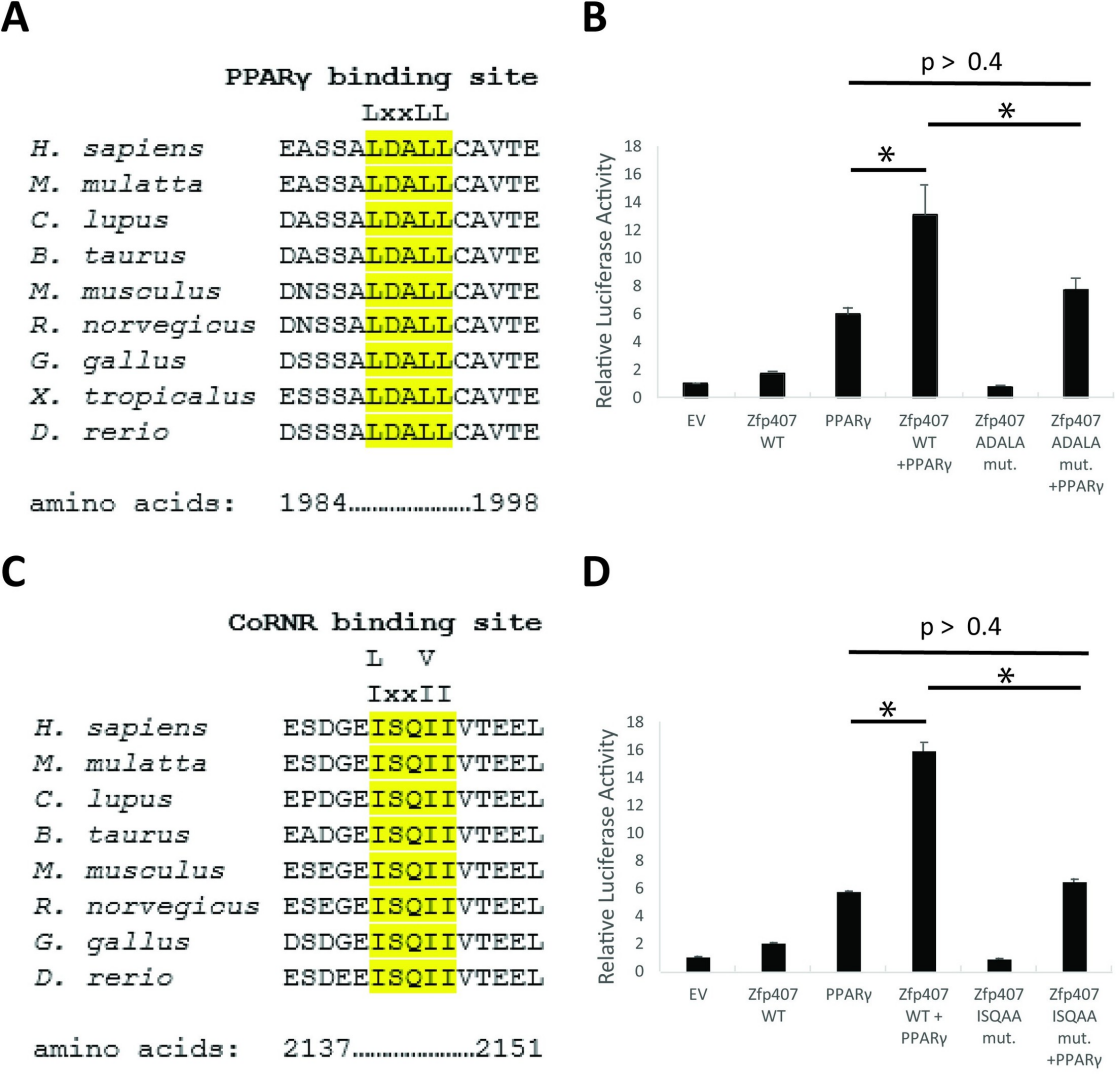
The PPARγ and RXR protein binding motifs in ZFP407 are required for transcriptional activation of PPARγ by ZFP407. Putative PPARγ and RXRα interaction motifs in ZNF407/ZFP407. (A) Amino acids 1984–1998 and (C) 2137–2151 of human ZNF407 are shown aligned with other species. Putative binding motifs are highlighted in yellow. (B,D) 293T cells co-transfected with the indicated plasmids and the PPRE PPARγ luciferase reporter.

Previous studies have identified consensus protein-protein interaction motifs required for binding to either PPARγ or RXR [42]. The PPARγ binding motif is comprised of an LxxLL amino acid sequence. The RXR binding motif, also referred to as a CoRNR motif, is comprised of an [I/L]xx[I/V]I amino acid sequence. These motifs are both found within the ZFP407 protein, consistent with our co-IP studies showing a direct interaction with the PPARγ/RXR protein complex (Fig 2A and 2B). The PPARγ binding motif is located at amino acids 1989–1993 in human ZNF407 and at amino acids 1980–1984 in the mouse ZFP407 (Fig 4A). The coRNR motif is found at amino acids 2142–2146 in the human ZNF407 and at amino acids 2140–2144 in the mouse ZFP407 (Fig 4C). Both the PPARγ binding motif and the coRNR motif demonstrate high levels of evolutionary conservation, suggesting an important function for their sequences, and consistent with their function of interacting with the PPARγ/RXR complex (Fig 4A–4C). In order to test the function of these two motifs in the transcriptional activity of ZFP407, the PPARγ binding motif was mutated from LDALL to ADALA, and the coRNR motif was mutated from ISQII to ISQAA, thereby disrupting the binding consensus sites for each. Whereas PPARγ co-transfection with a plasmid encoding wild-type (WT) ZFP407 more than doubled the transcriptional activity of PPARγ (Fig 4B–4D), co-transfection of a plasmid encoding a mutant allele of ZFP407 with either the PPARγ or RXR binding motif completely abolished the transcriptional effect of ZFP407 on PPARγ activity (Fig 4B–4D). This indicates the necessity of both motifs for transcriptional activation of PPARγ by ZFP407.

### ZFP407 does not modulate PPARγ activity through known PPARγ phosphorylation or SUMOylation sites

Transcriptional activity of PPARγ is moderated by post-translational modifications including phosphorylation and SUMOylation [43]. Phosphorylation of the serine 112 residue leads to either an increase or decrease of PPARγ activity depending on the context and the protein responsible for phosphorylation [43]. Phosphorylation of PPARγ’s serine 273 residue demonstrated no alteration of PPARγ’s adipogenic induction but altered a subset of PPARγ target genes expression that are commonly dysregulated in obesity including adiponectin and adipsin [44]. Additionally, SUMOylation of PPARγ at lysine 107 is known to decrease its transcriptional activity [45].

**Fig 5:**
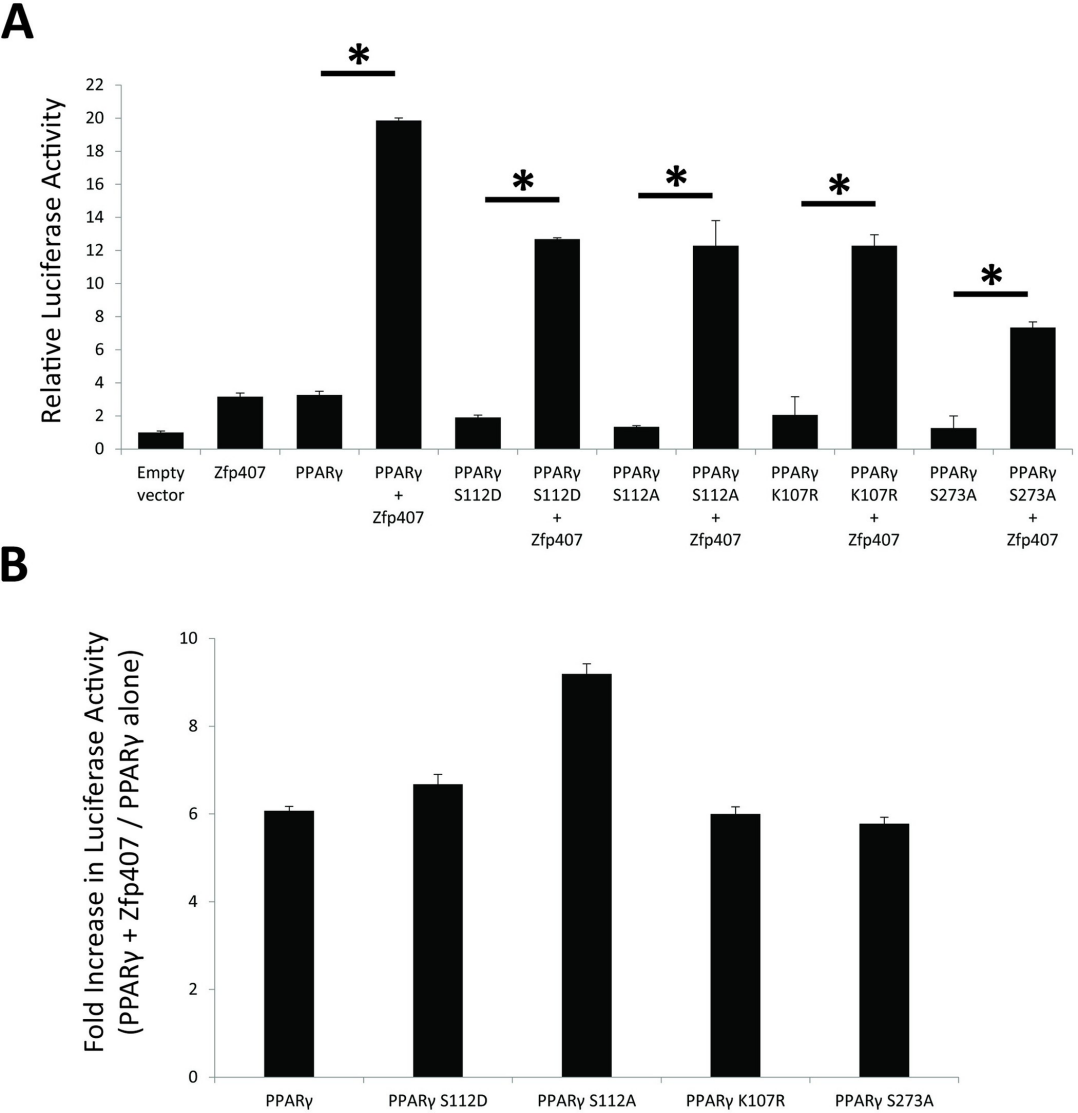
Mutation of PPARγ phosphorylation or SUMOylation sites doesn’t prevent synergistic transcriptional regulation by ZFP407. (A) Transfection of differentiated adipocyte 3T3-L1 cells with empty vector, ZFP407, WT PPARγ or PPARγ variants, or ZFP407 co-transfected with each of the PPARγ alleles. (B) Bars shown represent the relative fold-increase of PPRE-driven luciferase activity for co-transfection of the indicated PPARγ variant with ZFP407 when compared to transfection of the indicated PPARγ variant alone, derived from the data shown in panel A.

To test any potential effect of these modifications on ZFP407’s activation of PPARγ, PPARγ mutants were created with disruptions in each of these post-translational modified sites. The amino acids Serine 112 and Serine 273 were each mutated to alanine to prevent phosphorylation at these sites. Serine 112 was also mutated to aspartate to serve as a phosphomimic. Lysine 107 was mutated to arginine to prevent SUMOylation. Transfection of each of these PPARγ variants was shown to significantly increase PPRE luciferase reporter activity, but to a lesser extent than the WT PPARγ allele (Fig 5A). When co-transfected with ZFP407, the transcriptional activity of each of these mutant PPARγ alleles increased in a similar manner to that of WT PPARγ, suggesting that the effect of ZFP407 on PPARγ activity is independent of post-translational modifications at these sites (Fig 5B).

## Discussion

Immunostaining demonstrated co-localization of PPARγ and ZFP407 proteins in the nucleus (Fig 1A) when individually or co-transfected. Mutation of common PPARγ phosphorylation or SUMOylation sites did not prevent its regulation by ZFP407 (Fig 5). In addition, we’ve previously shown that ZFP407 deficiency does not alter PPARγ mRNA or protein levels [18]. These data suggest that ZFP407 controls the activity of PPARγ, but does so independently of post-translational modifications. Consistent with this hypothesis, we demonstrated that ZFP407 is a component of the PPARγ/RXR protein complex, as demonstrated by Co-IP with either an anti-RXR or anti-PPARγ antibody of endogenous proteins from 3T3-L1 adipocytes (Fig 2B). While it remains unknown if ZFP407 directly binds PPARγ or RXR, or binds indirectly via another cofactor, the presence of ZFP407 in this complex is consistent with a direct effect of ZFP407 on PPARγ signaling.

Cofactors directly interacting with PPARγ often contain at least one LxxLL motif contacting the coactivator binding groove in the ligand-binding domain of PPARγ [46]. ZFP407 contains an evolutionarily conserved LxxLL site (Fig 4A). When this site was mutated, it negated the synergistic effect of ZFP407 on PPARγ transcriptional activity (Fig 4B). Cofactors that directly interact with RXR often contain a CoRNR motif [I/L]xx[I/V]I [47]. ZFP407 contains an evolutionarily conserved CoRNR site (Fig 4C). When this site was mutated, it also negated the synergistic effect of ZFP407 on PPARγ transcriptional activity (Fig 4D). Collectively, this data suggests that ZFP407 directly binds PPARγ and RXR via these consensus binding motifs and that this interaction is required for transcriptional activation. The direct binding of ZFP407 could facilitate the interaction between PPARγ and RXR themselves, or facilitate additional cofactor recruitment.

The lone published ChIP-Seq study for ZFP407, a high-throughput analysis of transcription factors in a colon cancer cell line, demonstrated significant DNA-binding overlap between ZFP407 and RXR. The overlap between RXR and ZFP407 ranked in the top 15% of the >7,000 transcription factor pairs analyzed [48]. In addition, we determined that the majority of ZFP407 ChIP-Seq peaks overlapped with PPARγ ChIP-Seq peaks from a previously published dataset (Table 1) [31]. These overlapping peaks were most significantly enriched for the ZNF460 TF binding motif (Fig 3C). While little has been reported on ZNF460, either in general or specifically in adipocytes, a an intronic variant in *GLUT1* (rs841848) that has been associated with diabetic complications [49–51] and multiple glucose-related traits [52], is predicted to destroy a binding site for ZNF460 and has been associated with reduced *GLUT1* expression [53]. Other enriched motifs in PPARγ overlapping sites were ELK1, which has been linked to obesity as a target of miRNAs involved in adipogenesis, with decreased expression in obese visceral adipose tissue (VAT) [54], and TP73, which alters glycolysis within the liver via regulation of G6PD and other metabolic enzymes [55].

Among phenotypic manifestations of metabolic processes, body fat distribution has been shown to be an accurate predictor of risk for T2D and cardiovascular disease onset [56]. Excess adipocyte deposition in the upper body, nominally referred to as an “apple shape” body type, has been linked to dyslipidemia and insulin resistance [57]. The TF with the most enriched binding motifs among target sequences in the ZFP407 ChIP-seq analyses was CTCF, which is a ubiquitous TF that participates in transcription initiation and chromatin remodeling. CTCF promotes adipogenesis via its interactions with ATF4, with both proteins demonstrating co-localization in promoter regions tied to CEBPδ and PPARγ [58]. In adipose-derived stem cells sampled from patients demonstrating extreme phenotypes of “apple” and “pear shaped” body fat distributions, both *CTCF* and *ATF4* were downregulated in “apple shaped” body types, potentially linking them to the mechanisms driving body fat distribution in metabolic dysfunction [59].

Another significantly enriched TF binding motif among the ZFP407 ChIP-seq peaks corresponded to ZNF143, which regulates promoter-enhancer genomic loops bound to CTCF [60]. Furthermore, when utilizing both human and murine genome-wide ChIP-seq, ChIP-reChIP, and Hi-C approaches, ZNF143 binding of DNA was shown to facilitate the binding of CTCF to nearby genomic regions, thereby initiating transcription of target genes [60]. Notably, this binding was seen to have no impact on topologically associating domains (TADs), but rather was mostly relegated to promoter-enhancer interactions [60]. Deletion of *ZNF143* in hematopoietic progenitor cells and an inducible knockout in mice demonstrated diminished counts of mature white blood cells, believed to be on account of their inability to maintain their terminal phenotype [60]. In addition, the ZNF143 TF was previously posited as a biomarker for obesity-associated T2D [61]. Taken together, these studies suggest that ZNF143 and CTCF may operate together with ZFP407 by moderating promoter-enhancer genomic loops to regulate genes critical for adipocyte function and maintaining a healthy metabolic profile.

Among ZFP407 ChIP-seq peaks that did not co-segregate with PPARγ ChIP-seq peaks, nearly 50% of these target sequences contained at least one instance of the HSF4 binding motif. While HSF4 has not yet been directly linked to obesity or metabolic dysfunction, HSF1, another Heat Shock Transcription Factor is known to promote adipocyte browning via mitochondrial regulation [62]. Of the seven identified Heat Shock Transcription Factors, HSF1, HSF2, and HSF4 are the most structurally similar, with all three containing highly conserved N-terminal winged helix-turn-helix domains utilized for DNA binding, with the major dissimilarity being an extra leucine zipper in HSF1 and HSF2 [63]. Other enriched motifs among non-PPARγ overlapping peaks were the binding motif for TBX19 and the RARα/RXRγ heterodimer. *TBX19* variants have been linked to both obesity and neonatal hypoglycemia [64, 65]. Whereas TBX19 has been seen to play a role in regulation of WAT biology, RAR/RXR heterodimers can be linked to thermogenic remodeling of adipose tissue. In a small molecule drug screen identifying regulators of adipocyte browning, RAR/RXR TF heterodimers more specifically regulated BAT development and activation than even PPARγ [66], suggesting that ZFP407, when cooperating with non-PPARγ cofactors, may regulate BAT formation, consistent with the dramatic reduction of BAT in an adipocyte-specific *Zfp407* knockout mouse [20].

Protein interaction experiments demonstrated binding between ZFP407 and the PPARγ/RXR complex, establishing ZFP407 as a newly identified PPARγ cofactor and suggesting a direct effect on this signaling pathway. With interactions such as direct protein-protein agonism increasingly seen as targets for therapeutic intervention [67], identification of new cofactors, such as ZFP407, in the PPARγ pathway can reveal new drug targets for insulin resistance and T2D. While these studies clearly support the role of ZFP407 as an important cofactor regulating PPARγ/RXRα signaling, they have also provided insight into the other transcriptional pathways potentially regulated by ZFP407. The early embryonic lethality of ZFP07 knockout mice, which occurs around day e3.5 [20], and is considerably earlier than the mid-embryonic lethality of PPARγ deficient mice by day e10 [68], suggests key functions for ZFP407 beyond the regulation of PPARγ signaling that are required for organismal viability. There remains much to learn regarding the precise mechanism by which ZFP407 controls organismal development and adipocyte function, but these studies have provided new molecular insight into potential pathways and mechanisms by which ZFP407 controls the transcriptional networks that are critical for insulin sensitivity and organismal survival.

## Supporting information

S1 Table ChIP-seq peaks and motif enrichment hits. MACS2 identified peaks and motif enrichment as determined by HOMER in ZFP407 peaks segregated by overlap or non-overlap with PPARγ, CEBPα, and RXRα ChIP-seq peaks. Data included relates to motif searches considering all ZFP407 peaks as well as the top 1,000 peaks and JASPAR matched TF binding motifs for the top 1,000 peaks.
(XLSX)

S1 Raw images Original and unaltered images corresponding to blots for Figs 1B, 2A, and 2B. (PDF)
