# Supplementary material for: Molecular regulation of PPARγ/RXRα signaling by the novel cofactor ZFP407
Source: PLoS One. 2024 May 23;19(5):e0294003. doi: 10.1371/journal.pone.0294003 (PMC11115250; doi:10.1371/journal.pone.0294003)
Supplement: S1 Raw images — (PDF) [file pone.0294003.s002.pdf]

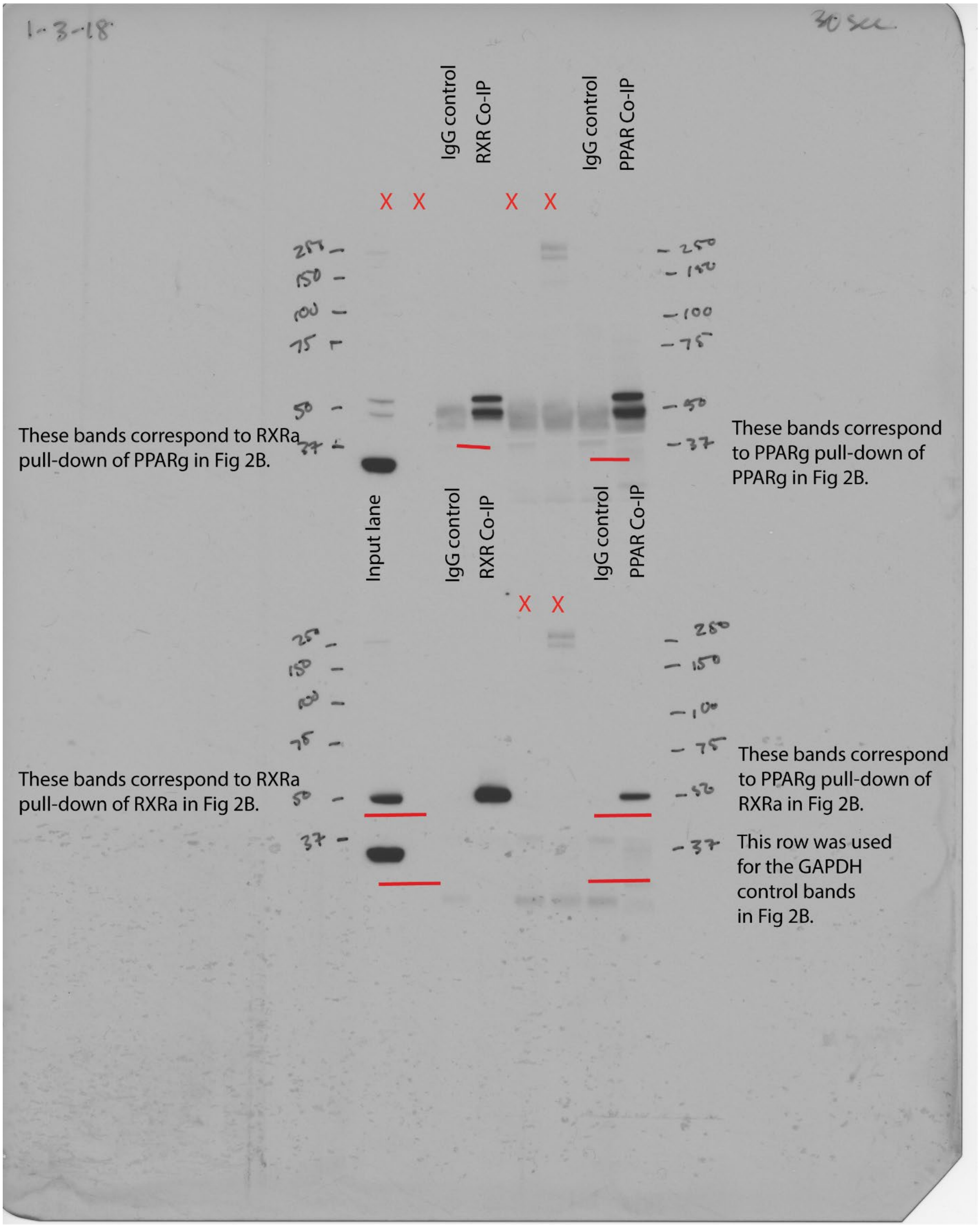

1-3-18
30 sec
IgG control
RXR Co-IP
IgG control
PPAR Co-IP
250
150
100
75
50
37
These bands correspond to RXRa pull-down of PPARg in Fig 2B.
These bands correspond to PPARg pull-down of PPARg in Fig 2B.
Input lane
IgG control
RXR Co-IP
IgG control
PPAR Co-IP
250
150
100
75
50
37
These bands correspond to RXRa pull-down of RXRa in Fig 2B.
These bands correspond to PPARg pull-down of RXRa in Fig 2B.
This row was used for the GAPDH control bands in Fig 2B.

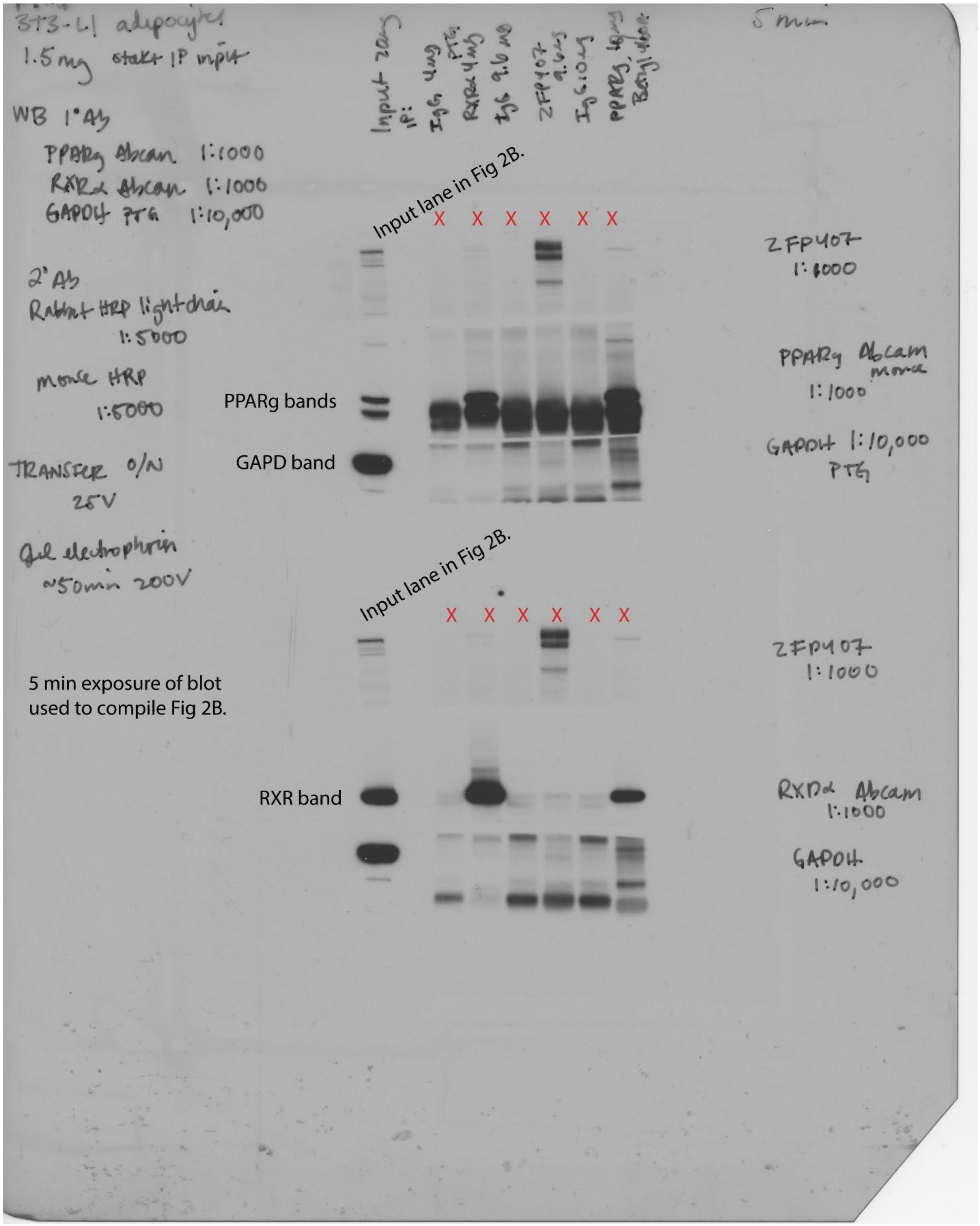
3T3-L1 adipocytes
1.5mg start IP input
WB 1°Ab
PPARg Abcam 1:1000
RXRα Abcam 1:1000
GAPDH PTG 1:10,000
2°Ab
Rabbit HRP light chain 1:5000
mouse HRP 1:5000
TRANSFER O/N 25V
Gel electrophoresis ~50min 200V
5 min
Input lane in Fig 2B.
PPARg bands
GAPD band
ZFP407 1:1000
PPARg Abcam mouse 1:1000
GAPDH 1:10,000 PTG
Input lane in Fig 2B.
5 min exposure of blot used to compile Fig 2B.
RXR band
ZFP407 1:1000
RXRα Abcam 1:1000
GAPDH 1:10,000

These PPAR bands were used to compile the panels in Fig 2A. (lanes 1 and 5)

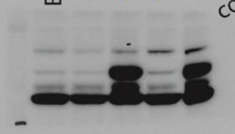

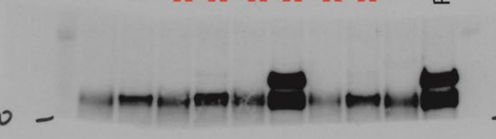

These PPAR bands were used for the CoIP panels in Fig 2A. (lanes 1,2,9,10)

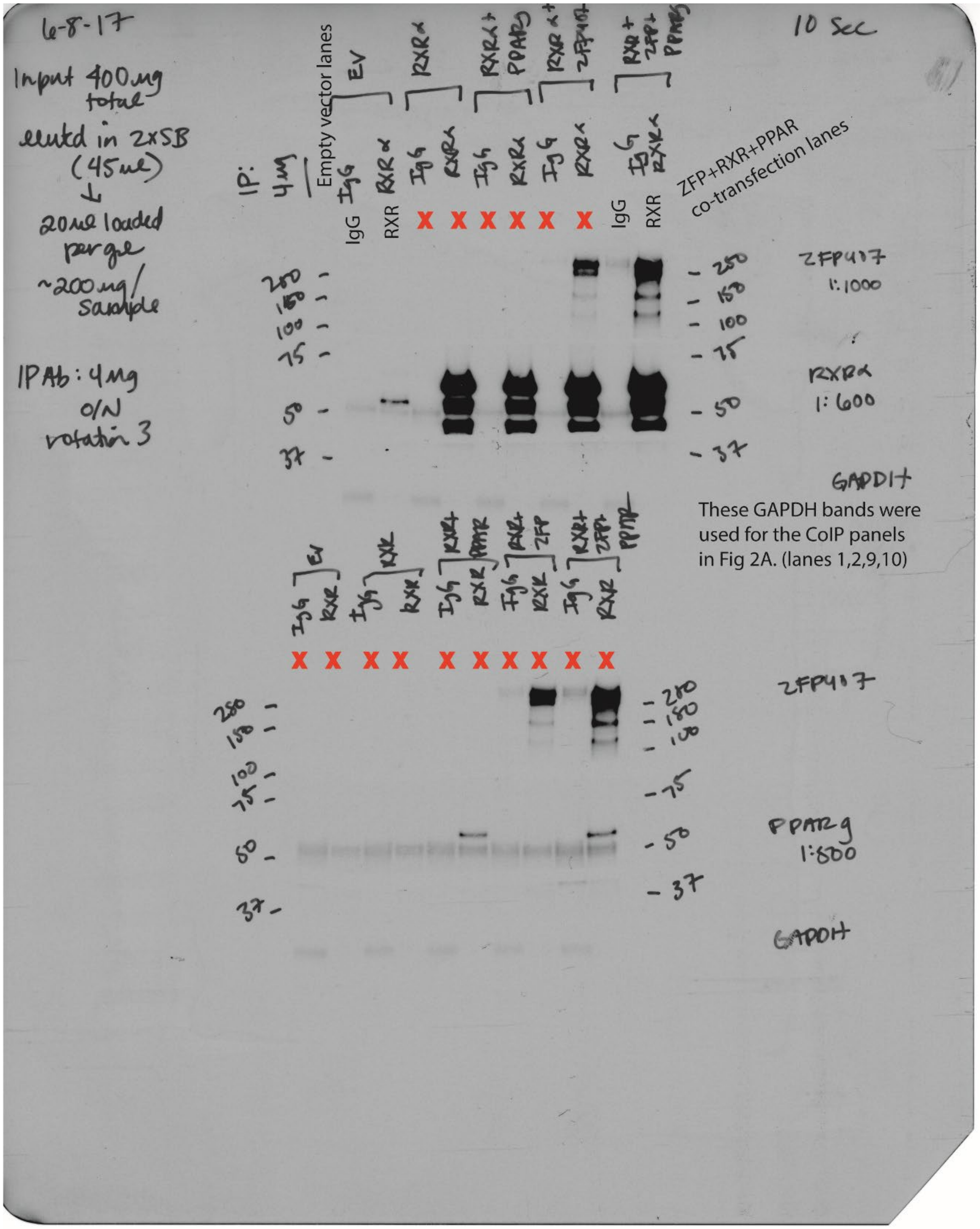

6-8-17
10 sec
Input 400ug total
eluted in 2xSB (45ul)
20ul loaded per gel
~200ug/sample
IP Ab: 4ug
O/N rotation 3
IP: 4ug
Empty vector lanes
EV
RXRα
RXRα+ PPARg
RXRα+ ZFP407
RXR+ ZFP+ PPARg
IgG
RXR
ZFP+RXR+PPAR co-transfection lanes
x x x x x x
250
150
100
75
50
37
ZFP407 1:1000
RXRα 1:600
GAPDH
These GAPDH bands were used for the CoIP panels in Fig 2A. (lanes 1,2,9,10)
x x x x x x x x x x
ZFP407
PPARg 1:800
GAPDH

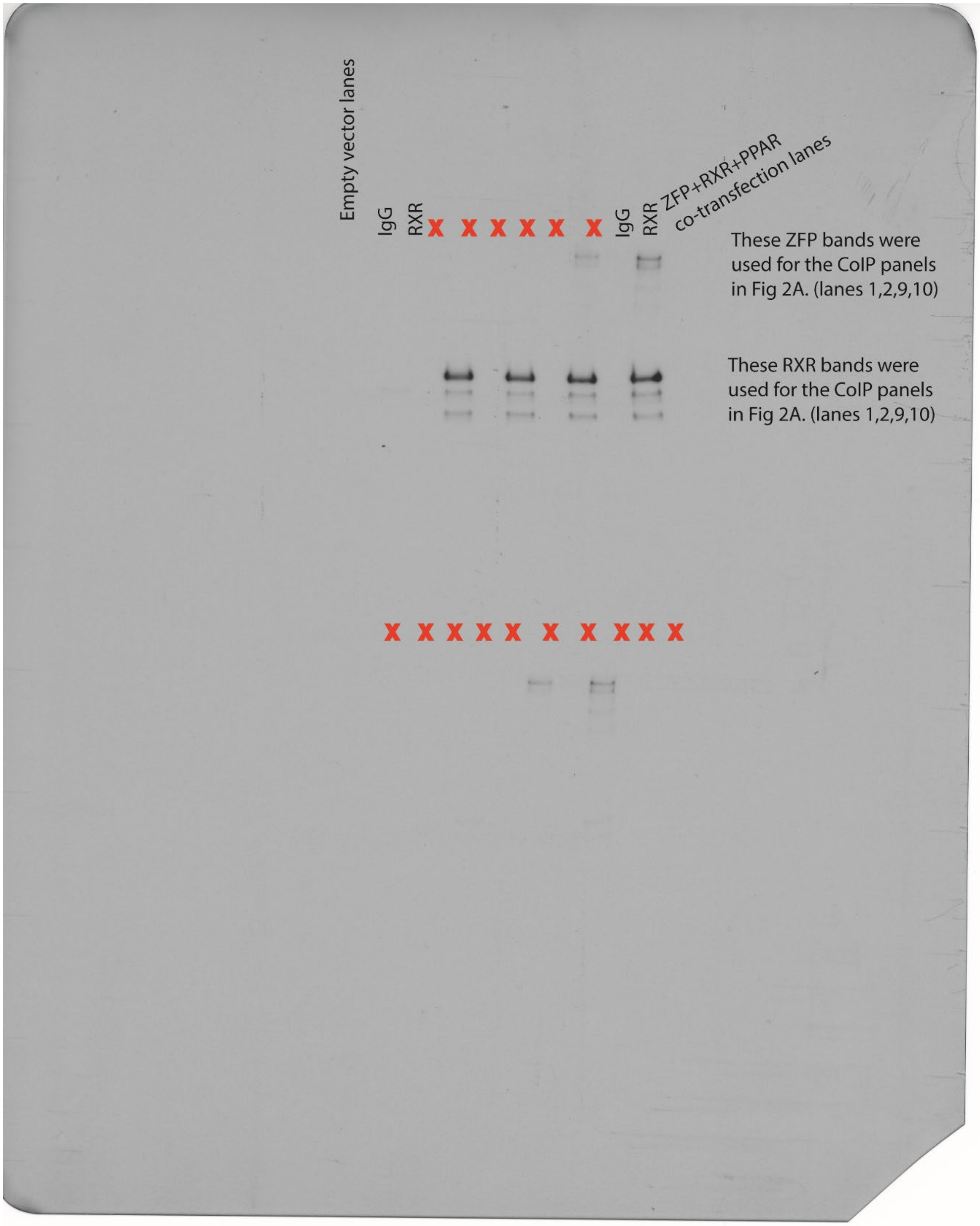
Empty vector lanes
IgG
RXR
IgG
RXR
ZFP+RXR+PPAR
co-transfection lanes
These ZFP bands were used for the CoIP panels in Fig 2A. (lanes 1,2,9,10)
These RXR bands were used for the CoIP panels in Fig 2A. (lanes 1,2,9,10)

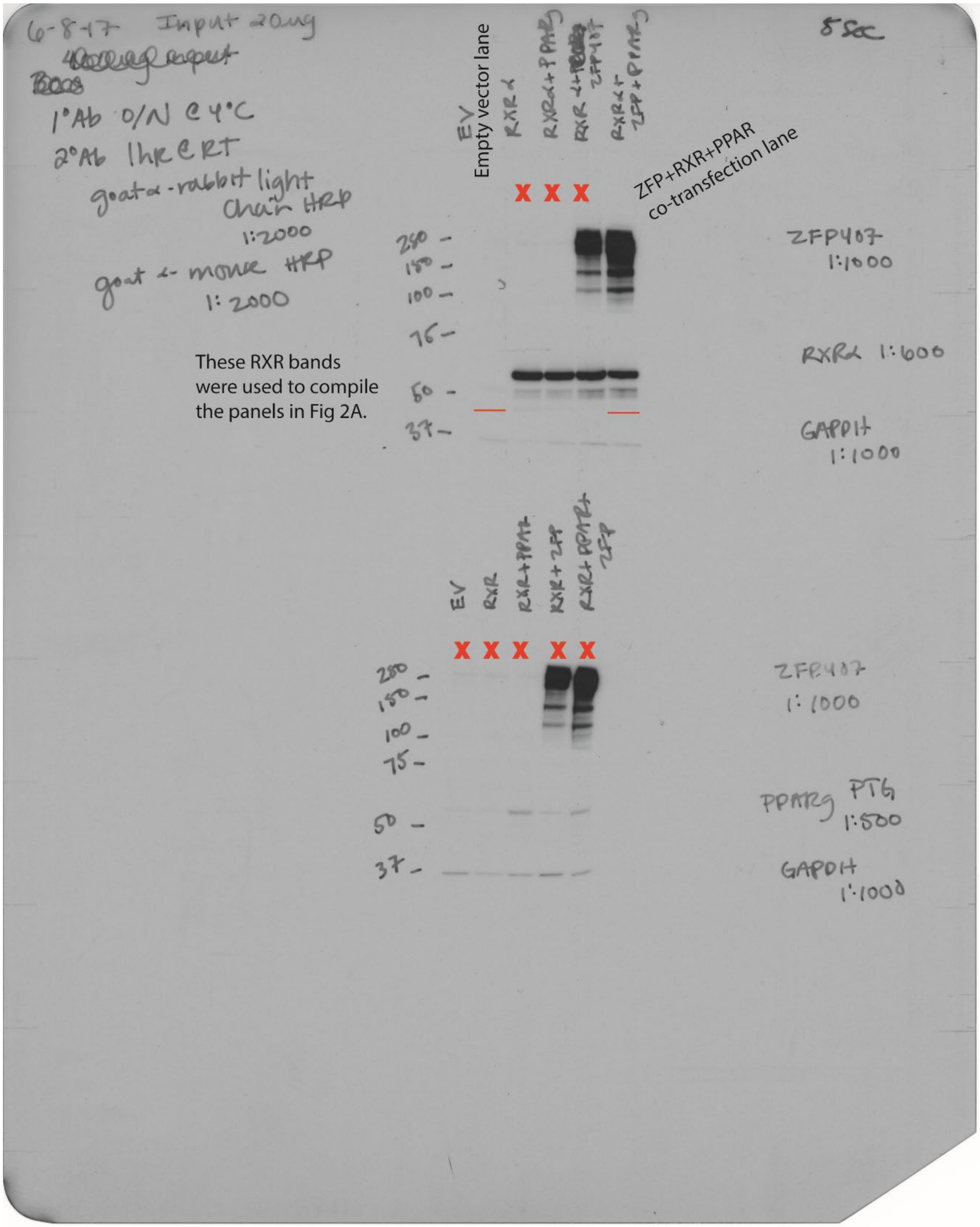
6-8-17 Input 20ug
1°Ab O/N @ 4°C
2°Ab 1hr @ RT
goat α-rabbit light chain HRP
1:2000
goat α-mouse HRP
1:2000
8 sec
EV
Empty vector lane
RXRα
RXRα+PPARγ
RXRα+ZFP407
RXRα+ZFP+PPARγ
ZFP+RXR+PPAR co-transfection lane
250
150
100
75
50
37
ZFP407 1:1000
These RXR bands were used to compile the panels in Fig 2A.
RXRα 1:600
GAPDH 1:1000
EV
RXR
RXR+PPARγ
RXR+ZFP
RXR+PPARγ+ZFP
250
150
100
75
50
37
ZFP407 1:1000
PPARg PTG 1:500
GAPDH 1:1000

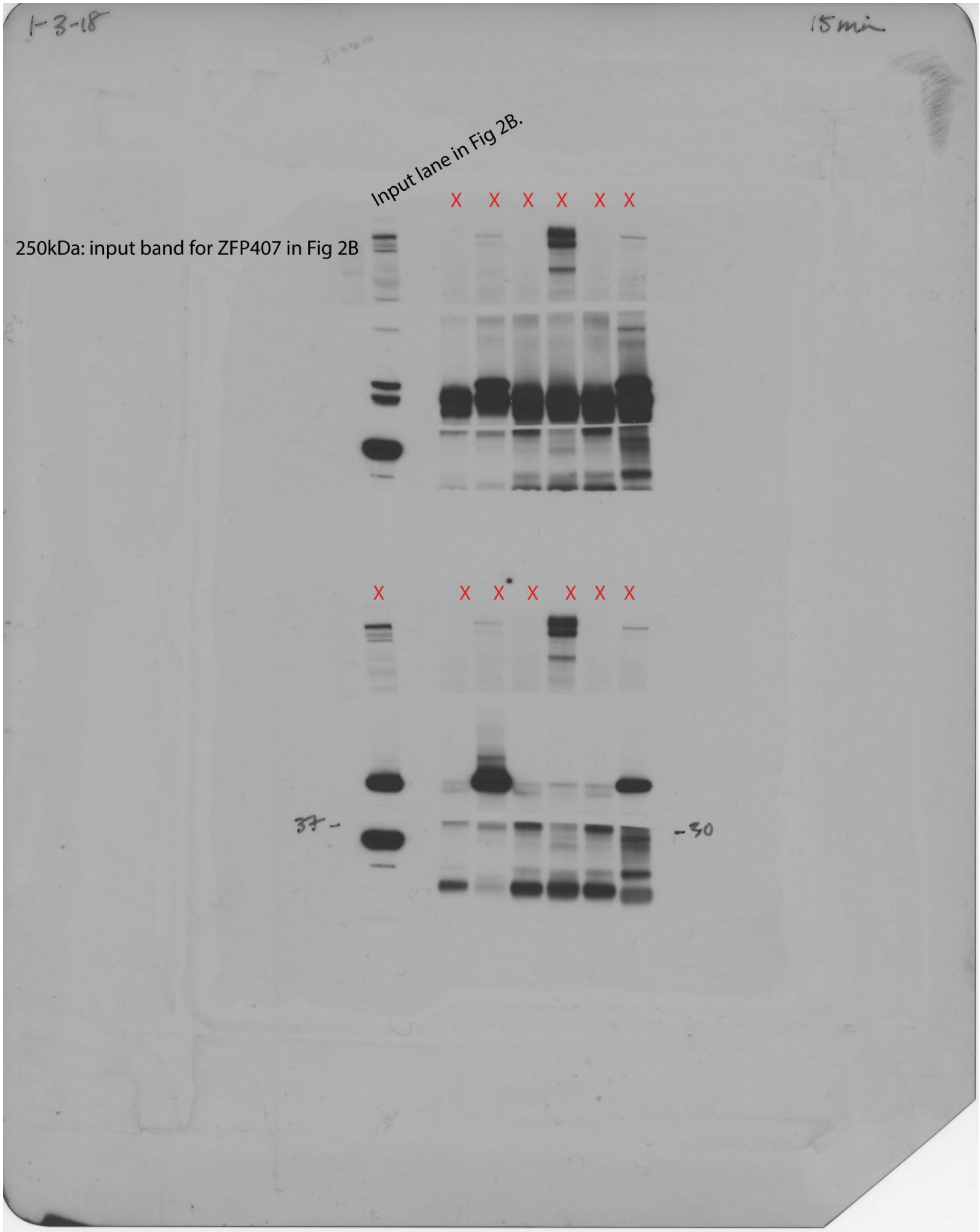
1-3-18
15min
Input lane in Fig 2B.
X X X X X X
250kDa: input band for ZFP407 in Fig 2B
X X X X X X X
37 -
- 30

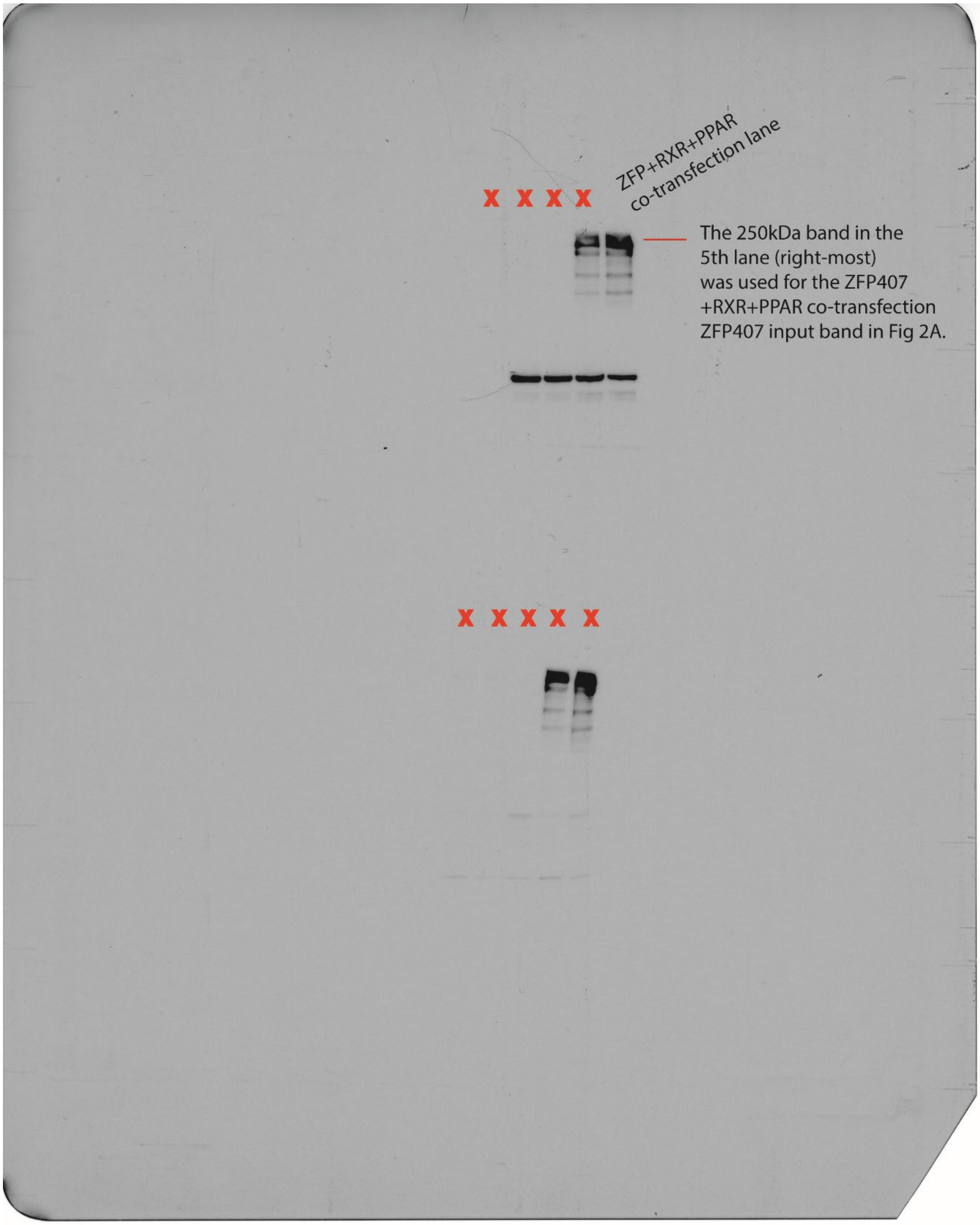
ZFP+RXR+PPAR
co-transfection lane
X X X X
The 250kDa band in the
5th lane (right-most)
was used for the ZFP407
+RXR+PPAR co-transfection
ZFP407 input band in Fig 2A.
X X X X X

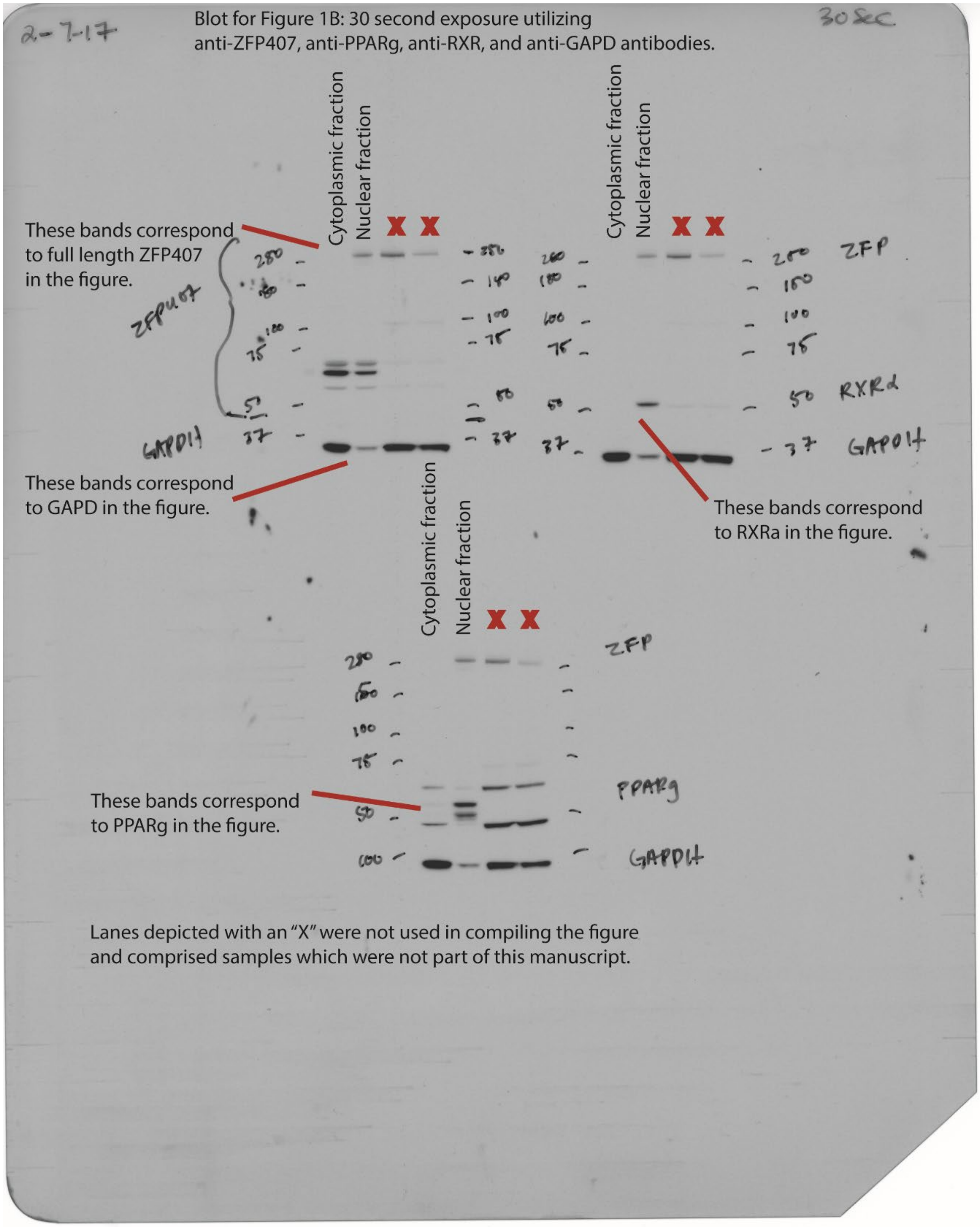
2-7-17
Blot for Figure 1B: 30 second exposure utilizing
anti-ZFP407, anti-PPARg, anti-RXR, and anti-GAPD antibodies.
30 Sec
Cytoplasmic fraction
Nuclear fraction
These bands correspond to full length ZFP407 in the figure.
ZFP407
GAPDH
These bands correspond to GAPD in the figure.
ZFP
RXRα
GAPDH
These bands correspond to RXRa in the figure.
PPARg
These bands correspond to PPARg in the figure.
Lanes depicted with an "X" were not used in compiling the figure and comprised samples which were not part of this manuscript.
